# Complex Left Atrial Appendage Morphology Is an Independent Risk Factor for Cryptogenic Ischemic Stroke

**DOI:** 10.3389/fcvm.2018.00131

**Published:** 2018-10-23

**Authors:** Indranill Basu-Ray, Deepthi Sudhakar, Gregory Schwing, Dominique Monlezun, Lucy Zhang, Sumit K. Shah, Deep Pujara, Kevin Ting, Nidal Abi Rafeh, Gholam Ali, Mark Cassidy, Kenneth Ellenbogen, Glen Levine, Wilson Lam, Nilesh Mathuria, Mohammad Saeed, Jared Bunch, Sheryl Martin-Schild, Michael Gold, Arash Aryana, Mehdi Razavi, Abdi Rasekh

**Affiliations:** ^1^Texas Heart Institute, Houston, TX, United States; ^2^St. Francis Hospital, Memphis, TN, United States; ^3^Department of Cardiology, Baylor College of Medicine, Houston, TX, United States; ^4^Department of Cardiology, Tulane University School of Medicine, New Orleans, LA, United States; ^5^Department of Mechanical, Aerospace and Nuclear Engineering, Rensselaer Polytechnic Institute, Troy, NY, United States; ^6^Department of Pathology, University of Arkansas for Medical Sciences, Little Rock, AR, United States; ^7^Division of Cardiology, Virginia Commonwealth University Medical Center, Richmond, VA, United States; ^8^Intermountain Heart Rhythm Specialists, Murray, UT, United States; ^9^Stroke Program, Department of Neurology, Tulane University School of Medicine, New Orleans, LA, United States; ^10^Department of Cardiology, Medical University of South Carolina, Charleston, SC, United States; ^11^Department of Cardiology and Cardiovascular Surgery, Mercy General Hospital, Dignity Health Heart and Vascular Institute, Sacramento, CA, United States

**Keywords:** left atrial appendage, left atrial appendage closure, cryptogenic stroke, atrial fibrillation, complex LAA morphology

## Abstract

**Importance:** Ischemic strokes pose a significant health burden. However, the etiology of between 20 and 40% of these events remains unknown. Left atrial appendage morphology may influence the occurrence of thromboembolic events.

**Design:** A retrospective cross-sectional study was conducted to investigate the role of LAA morphology in patients with atrial fibrillation (AF) and cardioembolic-associated stroke and patients with cryptogenic stroke without atrial fibrillation. LAA morphology is classified into two groups: (1) simple (chicken-wing) vs. (2) complex (non-chicken wing) based on transesophageal echocardiography (TEE) findings. In addition to the LAA morphology, left atrial parameters, including orifice diameter, depth, emptying velocity, and filling velocity, were collected for both groups. Mathematical, computational models were constructed to investigate flow velocities in chicken-wing and non-chicken wing morphological patterns to assess LAA function further.

**Findings:** TEE values for volume, size, emptying, and filling velocities were similar between simple and complex LAA morphology groups. Patients with cryptogenic stroke without coexisting AF were noted to have significantly higher rates of complex LAA morphology. Chicken-wing LAA morphology was associated with four-fold higher flow rate (kg/s) in computational simulations.

**Conclusions:** Complex LAA morphology may be an independent contributing factor for cryptogenic strokes. Further studies are warranted to investigate the mechanism involved in LAA morphology and thromboembolic events.

## Key points

**Question:** Is there an independent association between complex left atrial appendage morphology and incidence of cryptogenic ischemic strokes in patients without identifiable atrial fibrillation?
**Findings:** Of the 97 subjects meeting study criteria, subjects with complex LAA morphology had significantly increased odds of cryptogenic stroke as compared to those with simple LAA morphology.
**Meaning:** Left atrial appendage (LAA) morphology pattern investigation can be used as a technique to assess the risk of cryptogenic strokes and assist in devising newer therapeutic approaches for prevention and treatment.

## Introduction

Cryptogenic stroke is defined as cerebrovascular ischemia of unknown etiology. The specific etiologies of cryptogenic strokes remain obscure in almost 20–40% of cases, despite extensive investigations to elucidate the etiology (1). It is hypothesized that the most common event triggering a cryptogenic stroke is an embolus or emboli of cardiac origin (2). Atrial fibrillation (AF) is thus frequently suspected as the primary culprit for cryptogenic ischemic stroke. However, data from the Cryptogenic Stroke and Underlying AF (CRYSTAL AF) trial suggests that the rate of AF detection using implantable cardiac monitors in patients with cryptogenic stroke is only ~30% at 36 months (1). Hence, the remaining 70% of cases are presumed to be secondary to risk factors other than AF. Recent studies of subclinical AF have challenged the assumption that AF detected post stroke implicates an arrhythmic cause of the event (3). While numerous risk factors have been identified for cryptogenic stroke (1, 4, 5), only a few studies have investigated left atrial appendage (LAA) morphology as a potential culprit.

LAA has been traditionally divided into four morphological types: chicken wing, cactus, windsock, and cauliflower (6). The chicken wing morphological pattern is associated with higher velocities as compared to the cactus and cauliflower morphologies (7). Furthermore, it has been proposed that a single large lobe with high velocity (chicken-wing morphology) was inversely related to stroke events. On the other hand, smaller orifice, low velocity, and an increase in the number of trabeculation were directly correlated with stroke events. Cauliflower morphology indicated a higher risk of stroke (8). Also, the cauliflower type showed poor emptying and slow filling, creating more turbulence and increasing the risk of stroke/transient ischemic attack (9). The LAA morphology has been observed to be an independent marker for LAA flow velocity changes, and AF may not be the exclusive cause of cardioembolic events (10). The present study aims to investigate the relationship between LAA morphology and cryptogenic stroke using clinical and computational models.

## Methods

### Study population

The study population for these analyses was selected from the Tulane Stroke Registry after obtaining an institutional review board (IRB) approval for the study. Transesophageal echocardiography (TEE) was utilized to identify and evaluate patients for ischemic stroke at Tulane University Hospital from January 2009 to April 2016. Ischemic stroke was defined on a clinical basis and supported by neuroimaging with magnetic resonance imaging with diffusion-weighted imaging and/or repeat computed tomography scan. Those who met the inclusion criteria were included in the analysis. Inclusion criteria consisted of age greater than 18 years with a diagnosis of cardioembolic stroke with AF, and cryptogenic stroke without AF. Patients diagnosed with a stroke and included in the cryptogenic stroke group were screened for any current history of AF. Only those with no current and/or history of AF were included.

Additionally, all of the included patients were monitored with telemetry for the detection of AF throughout the acute admission as part of the evaluation of stroke etiology. All patients diagnosed with cryptogenic stroke were also thoroughly evaluated for secondary causes of ischemic stroke, including the presence of patent foramen ovale on TEE. Exclusion criteria consisted of mitral or aortic valve prostheses, poor TEE image quality, unavailability of TEE images and LAA ligation/exclusion. Stroke etiology was classified using the Trial of ORG 10172 in Acute Stroke Treatment (TOAST) causative classification system by vascular neurologists at Tulane University Hospital. For evaluation of the impact of LAA morphology on stroke, we identified patients with cryptogenic stroke as our subjects and patients with cardioembolic stroke as the comparison group.

### Transesophageal echocardiography

TEE assessed LAA morphology and function was used as it is considered to be the most widely used non-invasive modality to evaluate sources of stroke and suspected cardiac thromboembolism (11). A standard clinical TEE protocol (6-MHz multiplane transducer, Sonos 7500) was performed to assess for the presence of thrombus and various LAA parameters such as morphology, emptying and filling velocities (11). The LAA was visualized in the mid-esophageal view by rotating the imaging sector from 0 to 180°. The cursor was rotated to 120° with a counterclockwise rotation to obtain a transverse section and to approximately 90° to obtain a longitudinal section. Morphometric analysis was conducted by capturing still frames of the LAA progressing through the heart cycle, visualized in slow motion using Agfa HeartlabTM imaging software. Still frame images were collected at maximum, median and minimum areas in both views of the LAA and foreshortened when available.

**Figure 1:**
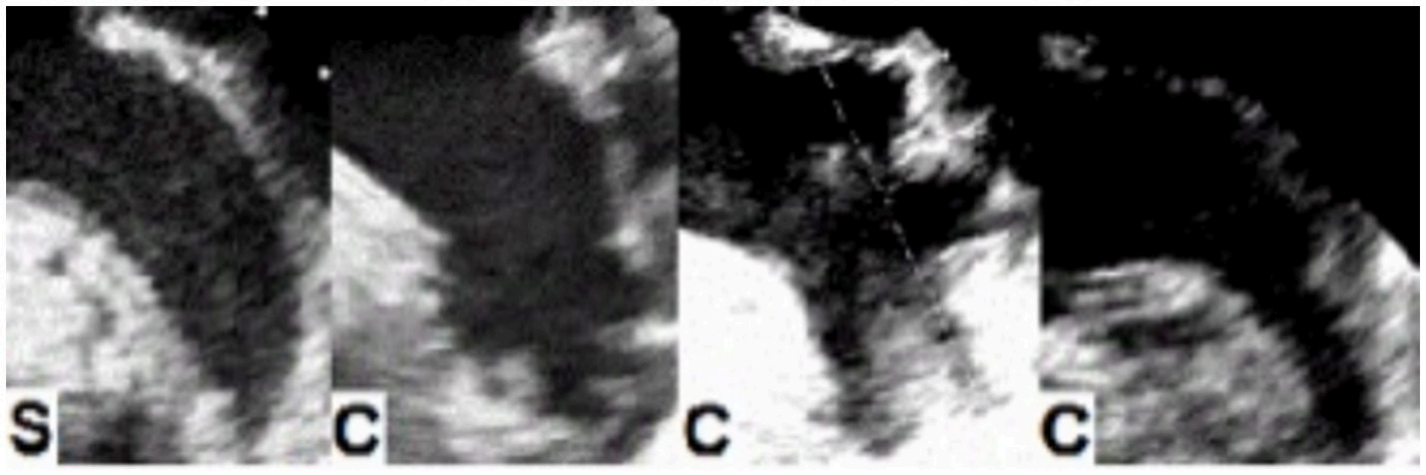
Transesophageal images of left atrial appendage (LAA) morphologies. Functional classification of LAA based on thrombogenicity. Left to right: S, simple LAA or SLA2M (chicken wing based on prior classifications); C, Complex LAA or CLA2M (includes chicken wing with pectinated walls and other complex morphologies including cauliflower, cactus and windsock).

The LAA as alluded to earlier has been classified into four morphological types based on their appearance: chicken wing, cactus, windsock, and cauliflower (Figure 1). However, this classification based on clinical appearance has very little clinical significance as far as stroke risk is concerned. Therefore we proposed a new functional classification (give the reference of our abstract). Two echocardiographers (independent expert imaging cardiologists) blinded to patient stroke etiologies were used to classify the TEE images as a “simple” or “complex” morphology. We defined “simple” as chicken wing morphology with clearly delineated endocardial walls. All the other morphologies were classified as “complex” including but not limited to: chicken wing with pectination, cauliflower, cactus, windsock, and other multi-lobed LAA morphologies. If no agreement could be achieved between the two observers, a consensus was reached using a third independent observer.

As noted above, other parameters besides morphology were also collected, such as the LAA orifice diameter, depth, and emptying and filling velocities. Orifice diameter was measured in the longitudinal view as the distance from the lateral ridge of the left superior pulmonary vein to the left coronary artery (12). Depth was measured from the midpoint of the orifice diameter to the farthest point from the center of the main lobe (13). The LAA blood flow velocities were measured by pulsed wave Doppler aimed at the proximal third of the LAA cavity. The biphasic wave amplitudes of 3–5 cardiac cycles measuring emptying flow velocity and filling flow velocity were averaged. This was achieved by taking a mean of the velocities from 3 consecutive cardiac cycles in patients with sinus rhythm. In those with AF, the cardiac cycles were often not related to the QRS complexes, and thus values from 5 consecutive cardiac cycles were averaged.

### Mathematical modeling

Mathematical modeling and computational simulations were created to investigate LAA function further, as previously reported (12). Briefly, 3-dimensional left atrium and LAA models with simple (chicken wing) and complex (non-chicken wing) morphologies were constructed based on physiologic measurements (Supplemental Images 1, 2). The parameters of LAA volume, LAA opening size, and LAA curvature were similar between the two groups. Simulations were conducted on the models to specifically investigate LAA flow rates (kg/s) and velocities in the chicken wing and non-chicken wing types at the LAA opening.

### Statistical analysis

Unadjusted associations among demographic, imaging and morphological variables were investigated. Categorical variables were compared using the Pearson chi-square or Fischer's exact test. Continuous variables were evaluated using an independent sample *t*-test or Wilcoxon rank sum test. Multivariable logistic regression was used to investigate the possible independent association between the stroke type and LAA morphology while adjusting for age and CHA2DS2-VASc score. Only these measures were adjusted for, due to the well-established association between an increase in the incidence of stroke with increasing age and higher CHADS2-VASc score. While some other variables may have some minimal effect on stroke, univariate, and multivariate analyses did not show a significant association between them Supplemental Tables 1, 2 and 3. Odds ratios (ORs) and 95% Confidence Intervals (CIs) were reported as fully adjusted results. Statistical significance was set at a two-tailed *p* < 0.05. Analyses were performed with STATA 14.0 (STATACorp, College Station, TX).

## Results

**Figure 2:**
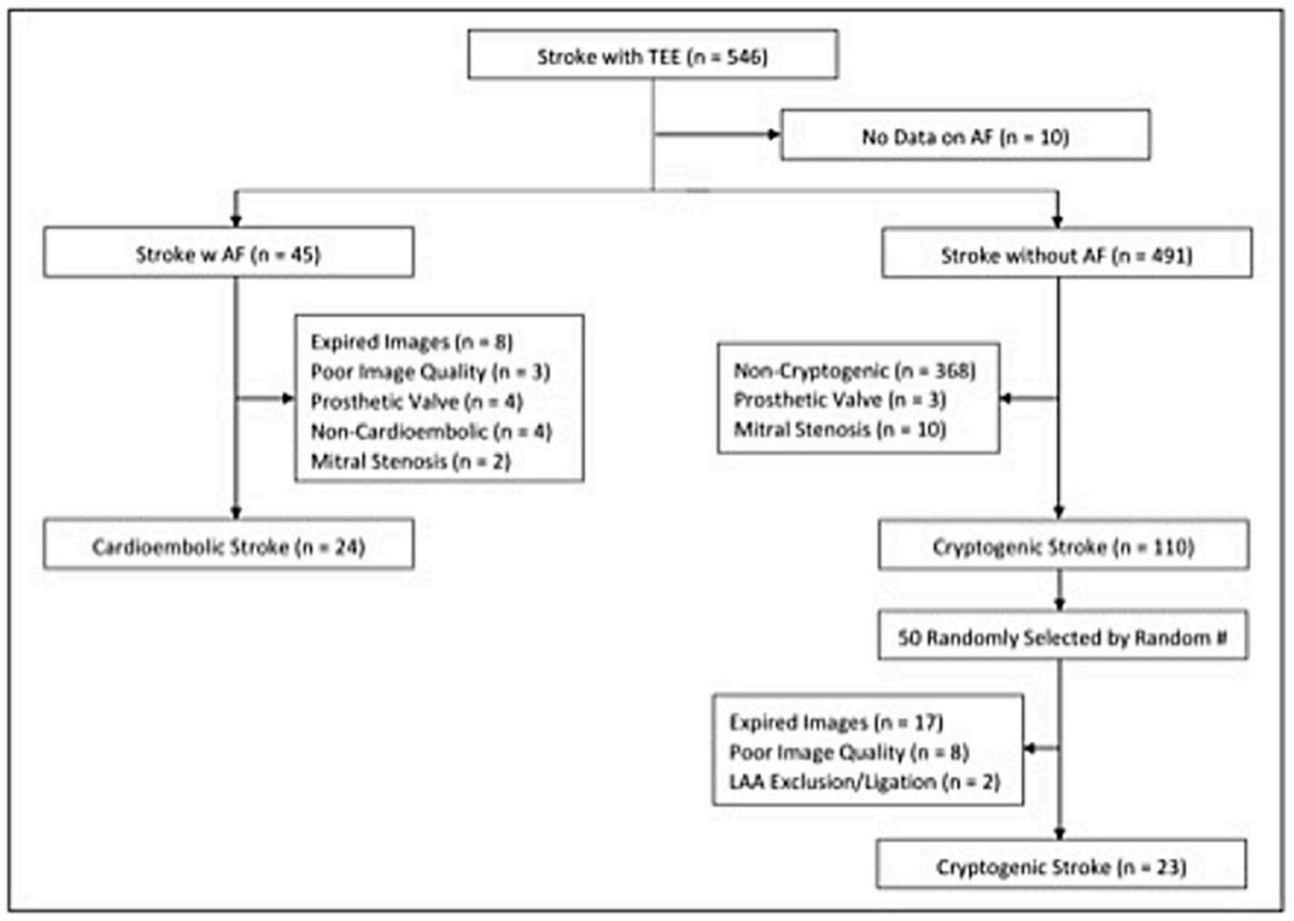
Study design and flowchart.

The Tulane Stroke Registry included 990 patients presenting with ischemic stroke between January 2009 and April 2016, of whom 546 patients had TEE reports available. Of these, 45 patients were identified as having an ischemic stroke with AF and underwent initial screening for the cardioembolic arm. Twenty-four of these were included in the final study population with breakdown shown in Figure 2. Of 110 patients with cryptogenic stroke, 50 patients were randomly selected by simple random sampling. Of these, 27 patients (54.0%) were excluded due to expired or poor quality images. Twenty-three patients (46.0%) with cryptogenic stroke with optimal TEE imaging met the final selection criteria. Thus, the study population included 24 patients (51.1%) with cardioembolic-associated stroke and 23 patients (48.9%) with cryptogenic stroke with identifiable LAA morphology. Of the 47 total patients, 24 patients (51.1%) had simple LAA morphology, whereas 23 patients (48.9%) were noted to have complex morphology. Table 1 compares patients with cardioembolic-associated stroke and cryptogenic stroke patients. Patients with cryptogenic stroke frequently demonstrated complex LAA morphology (15(65%) vs. 8(33%), *p* = 0.029). They also demonstrated higher age (70.4 ± 8.5 vs. 57.4 ± 10.6, *p* < 0.001) and CHA2DS2-VASc score (3.9 ± 1.7 vs. 2.7 ± 2.1, *p* = 0.029), a higher prevalence of tobacco use (13(56%) vs. 4(17%), *p* = 0.004) and lower prevalence of CHF (9(37%) vs. 1 (4%), *p* = 0.01). Fewer patients with cryptogenic strokes were on statins (5(22%) vs. 12(52%), *p* = 0.032).

**Table 1:** Parameter comparison between patients with cardioembolic associate stroke and cryptogenic stroke.

**Parameters**	**Cardioembolic stroke [*n* = 24]**	**Cryptogenic stroke [*n* = 23]**	***P*-value**
Age	57.43 ± 10.63	70.38 ± 8.52	0.0001^*^
Height	166.22 ± 22.63	173.22 ± 8.81	0.3365
Weight	84.94 ± 28.24	83.6 ± 18.37	0.965
BMI	38.93 ± 53.9	27.85 ± 5.55	0.809
CHA_2_DS_2_-VASc	2.65 ± 2.1	3.92 ± 1.69	0.029^*^
African American	11 (45.83)	13 (56.52)	0.464
Females	11 (45.83)	11(47.83)	0.891
Atrial Fibrillation	24 (100)	0 (0)	<0.001^*^
Prior stroke	8 (33.33)	9 (39.13)	0.679
Hypertension	20 (83.33)	15 (65.22)	0.154
Diabetes	8 (33.33)	10 (43.48)	0.474
Hyperlipidemia	11 (45.83)	11 (47.83)	0.891
Coronary artery disease	4 (16.67)	2 (8.7)	0.666
CHF	9 (37.5)	1 (4.35)	0.010^*^
CAD equivalents	3 (12.5)	1 (4.35)	0.609
Tobacco use	4 (16.67)	13 (56.52)	0.004^*^
Antiplatelet therapy	13 (54.17)	8 (34.78)	0.181
Anticoagulation therapy	4 (17.39)	0 (0)	0.109
Antihypertensive therapy	18 (75)	12 (52.17)	0.104
Statin therapy	12 (52.17)	5 (21.74)	0.032^*^
Complex LAA morphology	8 (33.33)	15 (65.22)	0.029^*^

* *Significant p-value*.

The baseline characteristics of patients with simple and complex LAA morphology are depicted in Table 2. The two groups demonstrated a non-significant difference in the mean age, gender, CHA2DS2-VASc score and other demographics including hypertension, heart failure, coronary artery disease, diabetes, hyperlipidemia, prior stroke, and antiplatelet, and anticoagulation therapy. However, patients with simple LAA morphology had significantly lower rates of cryptogenic stroke (8 patients (33%) as compared to the 15 patients (65%), *p* = 0.03) with complex LAA morphology. At the same time, the incidence of AF was higher in patients with simple LAA morphology when compared with patients with complex morphology (16 simple LAA patients (67%) vs. eight complex LAA patients (35%), *p* = 0.03).

**Table 2:** Baseline patient demographics.

**Variable**	**Simple LAA morphology [*n* = 24]**	**Complex LAA morphology [*n* = 23]**	***p*-value**
Age, y	65 ± 12	63 ± 12	0.60
Height, cm	167 ± 10	172 ± 10	0.59
Weight, Kg	84 ± 24	85 ± 24	0.67
BMI, kg/m^2^	38 ± 54	29 ± 7	0.96
CHA_2_DS_2_-VASc	3.4 ± 1.9	3.2 ± 2.1	0.68
African American, *n* (%)	11 (46%)	13 (56%)	0.46
Females, *n* (%)	11 (46%)	11 (48%)	0.89
Atrial fibrillation, *n* (%)	16 (67%)	8 (35%)	0.03^*^
Prior stroke, *n* (%)	9 (38%)	8 (35%)	0.85
Cryptogenic stroke, *n* (%)	8 (33%)	15 (65%)	0.03^*^
Hypertension, *n* (%)	19 (79%)	16 (70%)	0.45
Diabetes, *n* (%)	8 (33%)	10 (43%)	0.47
Hyperlipidemia, *n* (%)	11 (46%)	11 (48%)	0.89
Coronary artery disease, *n* (%)	3 (12%)	3 (13%)	0.96
Heart failure, *n* (%)	6 (25%)	4 (17%)	0.72
Coronary artery disease equivalent, *n* (%)	2 (8%)	2 (9%)	1.00
Tobacco use, *n* (%)	7 (30%)	10 (43%)	0.36
Antiplatelet therapy, *n* (%)	12 (50%)	9 (39%)	0.45
Anticoagulation therapy, *n* (%)	3 (12%)	1 (5%)	0.61
Antihypertensive therapy, *n* (%)	15 (62%)	15 (65%)	0.85
Statin therapy, *n* (%)	10 (42%)	7 (32%)	0.85
Moderate/severe mitral regurgitation, *n* (%)	8 (33%)	8 (39%)	0.61

* *Significant p-value*.

As demonstrated in Table 3, the imaging parameters obtained by TEE were similar when comparing simple and complex LAA morphologies, including peak LAA filling and emptying velocities, LAA orifice diameter, and LAA depth. However, simple LAA morphology exhibited a non-significant trend toward a higher left atrial volume index as compared to complex LAA morphology (46 ± 26 vs. 35 ± 17 mm^3^, *p* = 0.08).

**Table 3:** Transesophageal echocardiography (TEE) imaging parameters.

**Parameter**	**Simple LAA morphology [*n* = 24]**	**Complex LAA (1) morphology [*n* = 23]**	***p*-value**
Pulmonary artery pressure, mmHg	40 ± 13	41 ± 16	0.82
Left atrial size, mm	52 ± 61	42 ± 26	0.73
Left atrial volume index, mm^3^	46 ± 26	35 ± 17	0.08
Left ventricular ejection fraction, %	47 ± 16	55 ± 8	0.13
Left ventricular size, mm	75 ± 18	108 ± 81	0.31
Peak LAA filling velocity, cm/s	36 ± 20	36 ± 15	0.95
Peak LAA emptying velocity, cm/s	38 ± 21	44 ± 21	0.43
LAA orifice diameter, mm	20 ± 6	18 ± 6	0.14
LAA depth, mm	37 ± 12	35 ± 13	0.36

Identification of variables of risk associated with cryptogenic vs. cardioembolic-associated stroke was also attempted. Based on a multivariate logistic regression analysis, age [OR: 0.85 [95% CI: 0.75–0.95], *p* = 0.004] and complex LAA morphology [OR: 5.00, [95% CI: 1.05–23.73], *p* = 0.04] were identified to be independently associated with the presence of cryptogenic stroke even after adjusting for the CHA2DS2-VASc risk score (Table 4).

**Table 4:** Multivariable logistic regression model for association between cryptogenic stroke and complex left atrial appendage (LAA) morphology adjusted for age and CHA_2_DS_2_-VASC score.

**Variable**	**Odds ratio**	**Standard error**	***p*-value**	**[95% Confidence Interval]**	
Age	0.85	0.05	0.004^*^	0.75	0.95
CHA_2_DS_2_-VASc score	0.96	0.21	0.85	0.62	1.47
Complex LAA morphology	5.00	3.97	0.04^*^	1.05	23.7

* *Significant p-value*.

**Figure 3:**
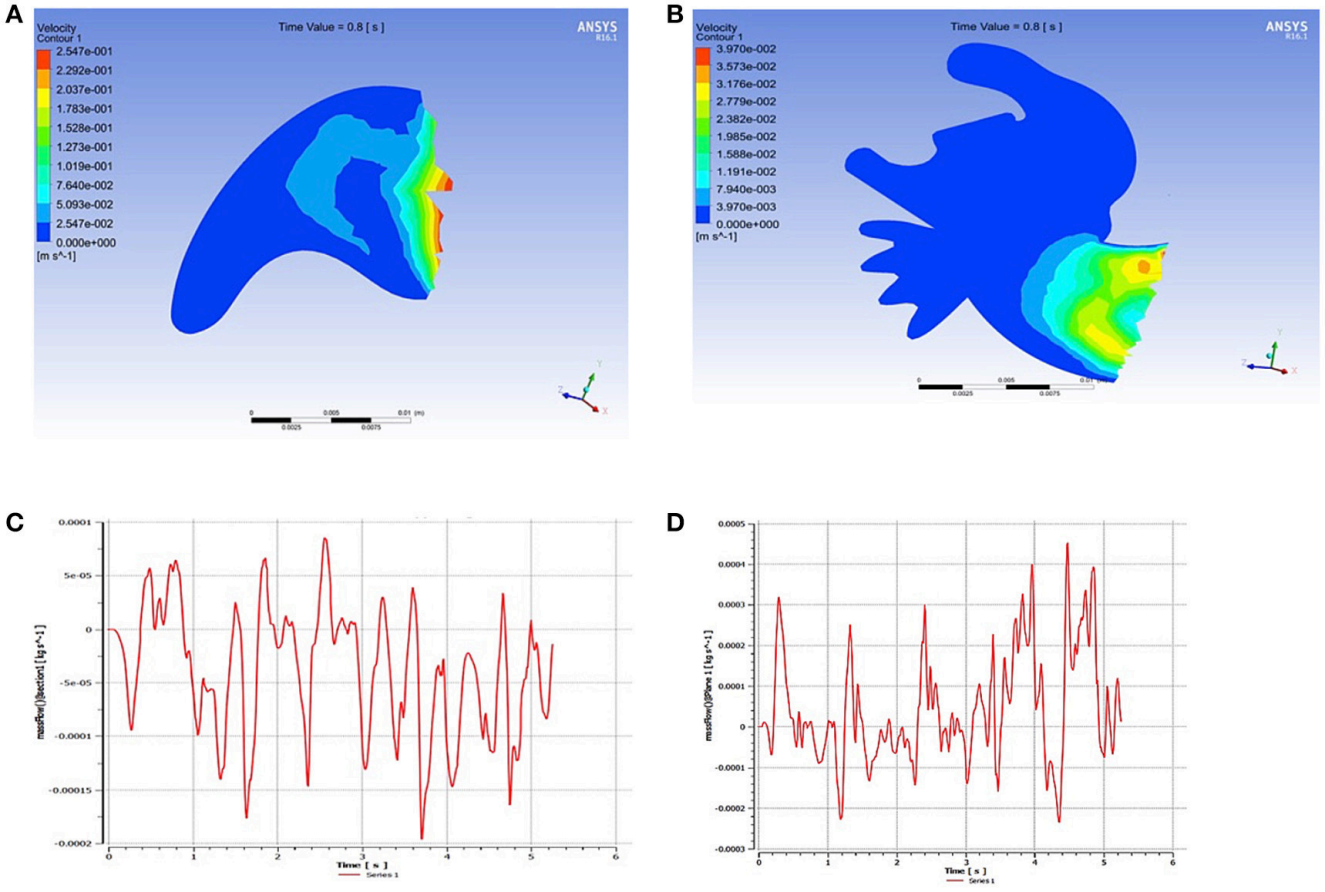
Mathematical modeling of flow velocities inside the left atrial appendage (LAA). Flow velocities of the simple **(A)** and complex **(B)** LAA morphologies. The chicken wing LAA morphology **(C)** is associated with 4-fold higher flow rate (kg/s) at the LAA opening as compared to the non-chicken wing morphology **(D)**.

**Figure 4:**
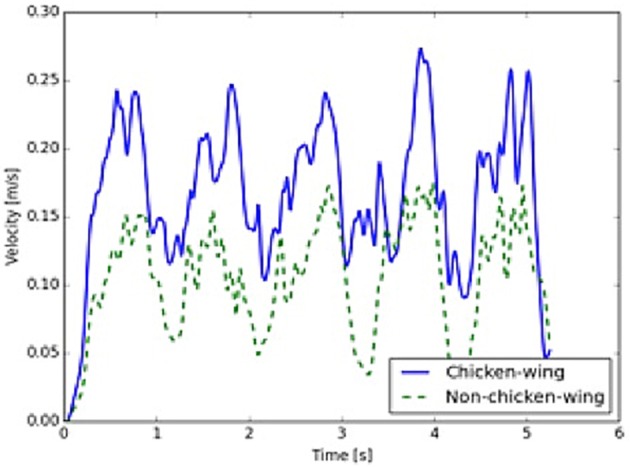
Left atrial appendage (LAA) opening velocities. Velocities at the center of the LAA orifice are higher in the chicken wing (solid, blue) as compared to the non-chicken wing (dashed, green) morphology (time measured over 5 cycles).

Furthermore, a comparison of LAA using mathematical modeling simulations demonstrated that flow rates (kg/s) were 4 times higher in the chicken wing LAA as compared to non-chicken wing morphologies (Figure 3, Supplemental Videos 1, 2). The maximal LAA orifice velocity, as measured at the orifice center, was also found to be higher in the chicken wing morphology (Figure 4).

## Discussion

Etiological factors for cryptogenic stroke remain unknown. Despite routine evaluation, almost 20 to 40% of stroke cases are classified as cryptogenic stroke (1). Atrial Fibrillation is probably not the cause in around 70% of this cohort as determined by an implantable loop recorder. A noteworthy finding in this study is the significantly higher prevalence of complex LAA morphology observed among the cohort of patients with cryptogenic stroke. On the other hand, parameters including size, volume, filling and emptying velocities remain almost identical throughout. Simulations of LAA function in computational models further supported these findings by demonstrating low-flow velocities within the complex LAA morphology. As such, it is hypothesized that the trabeculations within the complex LAA morphology may induce rheological changes that may serve as a nidus for thrombosis and promote thromboembolism, particularly in the setting of lower flow velocities and stasis.

Results from the Warfarin-Aspirin Recurrent Stroke Study (WARSS) trial showed no difference between warfarin and aspirin in secondary stroke prevention in patients without AF (13). Another study stipulated similar findings after investigating study subjects over a two-year period. It was concluded that there was no difference between warfarin and aspirin in recurrent stroke prevention and their safety profile in causing major hemorrhage (14). However, a *post-hoc* analysis demonstrated that warfarin is superior in those with an elevated N-terminal pro-brain natriuretic peptide (pro-BNP), a biomarker that is believed to reflect LAA function (15). Given that AF is identified only in a minority of patients with cryptogenic stroke who undergo long-term cardiac rhythm monitoring (1), it is plausible that LAA dysfunction and/or morphology could confer additional risk of thromboembolism in the absence of AF. Indeed, there have also been sporadic reports on the occurrence of LAA thrombus even in patients who remain in sinus rhythm.

For instance, in a consecutive series of patients with stroke in the absence of significant carotid arterial stenosis, Labovitz (16) found that patients with unexplained cerebral ischemia had detectable abnormalities on TEE. Left ventricular enlargement and aortic plaque were detected on TEE and were at an increased risk of recurrent stroke when on monotherapy with aspirin. Similarly, Vigna et al. (17) reported that systemic emboli occurred in patients with dilated cardiomyopathy. Left atrial and ventricular thrombi are considered as an etiologic factor for systemic emboli in patients with dilated cardiomyopathy. On the other hand, a stronger association was found between left atrial thrombus and stroke as compared to left ventricular thrombus and stroke. These and other reports have suggested that the LAA may perhaps even serve as a source of thromboembolism in the absence of AF (18).

The LAA morphology has been historically classified into four types (chicken wing, windsock, cauliflower, and cactus) (6). Specific anatomical morphology has been observed to be an independent marker for stroke and LAA is the most common site in the left atrium for thrombus formation. LAA flow velocities were higher in chicken wing morphology as compared to the cactus or the cauliflower morphologies (19). Besides, the number of LAA lobes adds complexity and also is a risk factor for thrombus formation (19). A study by Di Biase et al. (20) concluded that patients with a chicken wing LAA morphology had a significantly lower risk of prior thromboembolism, whereas the cauliflower morphology appears to be most frequently associated with thromboembolism (20, 21). The wide variability of the LAA anatomical types and the ambiguous definition of the morphologic types led the morphological classification to be labeled as either “simple” or “complex.” In the definition used in this study, a “simple” LAA morphology corresponded to any LAA with a single lobe and discrete walls without crevices (i.e., a classic chicken wing). On the other hand, a “complex” morphology was applied to LAA morphologies with multiple lobes (cauliflower, cactus, or windsock types) or a single lobe with irregular walls and small or large crevices (a chicken wing morphology with significant trabeculation) (Figure 2). Our nomenclature was based on the observation that extensive LAA trabeculation has been proposed to influence stasis and thrombus formation (8).

The association of complex LAA morphology and stroke was much more apparent in patients with cryptogenic events. We hypothesize that AF overwhelmingly dominates as a cause of cardioembolic stroke, irrespective of the presence or absence of other risk factors. Furthermore, the presence of AF and its progression also is associated with structural changes on the LAA (22, 23). The observation that the presence of diastolic dysfunction obviated this significance is understandable as substantial diastolic dysfunction is associated with increased incidence of AF and morphological and functional changes of the left atrium (24). Thus, it may be deduced that complex LAA morphology by itself may serve as a significant risk factor for thromboembolism leading to cryptogenic stroke in the absence of AF or early in its disease course. However, in the presence of AF, in general, the overwhelming mechanism of stroke is cardioembolic irrespective of LAA morphology.

Given the severe morbidity of cryptogenic strokes, it is particularly essential to determine the various risk factors, which in turn may influence clinical management and outcomes. Our results suggest that LAA morphology may, in fact, play an important role in cryptogenic stroke. The randomized investigation of a larger cohort may reveal a role for anticoagulation in cryptogenic stroke prevention. Moreover, these results may provide evidence to explore the possibility of LAA closure for those at the highest risk of cryptogenic stroke recurrence with contraindication to anticoagulation.

## Limitations

This study has several limitations. This is a pilot study based on preliminary data derived from only a cohort of stroke patients to prove a hypothesis. However, the findings are significant, as it involves millions of patients who get a recurrent stroke with unknown etiology. Lacuna of etiological knowledge has also precipitated a hiatus in therapy, and appropriate prevention protocol is still evolving. For example, a patient with cryptogenic stroke and complex LAA may be a candidate for anticoagulation therapy rather than dual antiplatelet therapy for secondary prevention as is the current norm. Based on these findings, an association between the complex LAA morphology and cryptogenic stroke is proposed. Also, patients with cryptogenic stroke and coexisting AF were not included in the study to prevent confounding of the results. Derivation of a causal pathway between stroke and complex LAA morphology would require a more detailed and comprehensive study, particularly involving both stroke and non-stroke cohorts. First, the investigation is limited by its retrospective, cross-sectional study design, along with a small sample size and single-center study cohort.

Second, exclusion of etiologies other than cryptogenic and cardioembolic-associated stroke prevented a more detailed analysis of the capacity in which LAA morphology may influence different stroke etiologies. However, it should be emphasized that these are the most clinically plausible etiologies to be affected by cardiogenic factors and among the most commonly noted on TEE among patients with acute stroke.

Finally, the presence of AF in this study could have been underestimated because of the lack of a long-term follow-up. Some patients may have developed AF following the time frame of the current study. However, as evidenced by the CRYSTAL-AF study, patients who were delineated as having a stroke after a month of monitoring were only a minority. The rate of atrial fibrillation detection even after the 12-month follow-up was only 12.4% (2). Our cohort has been defined as one having no history of atrial fibrillation, along with cardiac monitoring in the hospital for at least 7 days with no evidence of Atrial Fibrillation. While this could indeed have overestimated the number of cryptogenic strokes, it is still a starting point by which most of this type of stroke is diagnosed, but could be further confirmed through long-term monitoring.

## Conclusion

In conclusion, the study results suggest that complex LAA morphology is an independent risk factor for stroke. The aberrant rheological phenomenon induced by complex LAA morphology could precipitate “cryptogenic” stroke as this study shows. In addition to that, it can also be an ancillary factor playing an additive role in the etiologic factors for cryptogenic stroke. Simulations of LAA function in computational models further support these findings by demonstrating low-flow velocities within complex LAA anatomy. As this is a pilot study proposed to entertain a proof of concept, future studies are warranted to more clearly investigate the suggested association between LAA morphology and the risk of thrombus formation. Irrevocable proof of this concept by rigorous scientific data in the future might help to elucidate a better preventive protocol in the clinical management and prevention of stroke. The role of long-term anticoagulation and LAA closure in patients with complex LAA morphology will then be better defined to prevent this disorder.

## Author contributions

IB-R concept; DS, SS, and DP drafting article; GS, DM, LZ, KT, NR, GA, MC, KE, GL, WL, NM, MS, JB, SM-S, MG, AA, MR, and AR various inputs including critical revision of the article, methods, and interpreting study data.

### Conflict of interest statement

The authors declare that the research was conducted in the absence of any commercial or financial relationships that could be construed as a potential conflict of interest.

## Supplementary material

Supplemental Table 1 Univariate analyses to assess the association between cryptogenic stroke and left atrial appendage (LAA) filling velocity.

Supplemental Table 2 Univariate analyses to assess the association between cryptogenic stroke and LAA emptying velocity.

Supplemental Table 3 Multivariate analyses to assess the association between cryptogenic stroke and variables like age, CHA2DS2-VASc score, complex left atrial appendage (LAA) morphology, LAA filling velocity and LAA emptying velocity.

Supplemental Video 1 Video S1- Flow velocities in the simple/chicken-wing LA.

Supplemental Video 2 Video S2- Flow velocities in the complex/non chicken-wing LAA.

Supplemental Image 1 Flow velocities in simple/chicken wing LAA.

Supplemental Image 2 Flow velocities in complex/non chicken wing LAA.

## Abbreviations

LAA: Left Atrial Appendage
AF: Atrial Fibrillation
TEE: Transesophageal Echocardiography
TTE: Transthoracic Echocardiography
CT: Computed Tomography
CRMI: Cardiac Magnetic Resonance Imaging
TEs: Thromboembolic Events
OR: Odds Ratio
CI: Confidence Interval.
