# Supplementary material for: Complex Left Atrial Appendage Morphology Is an Independent Risk Factor for Cryptogenic Ischemic Stroke
Source: Front Cardiovasc Med. 2018 Oct 23;5:131. doi: 10.3389/fcvm.2018.00131 (PMC6232927; doi:10.3389/fcvm.2018.00131)
Supplement: Supplemental Table 1 — Univariate analyses to assess the association between cryptogenic stroke and left atrial appendage (LAA) filling velocity. [file Table_1.DOCX]

**Supplemental tables:**

**Supplemental table 1: Univariate analyses to assess the association between cryptogenic stroke and left atrial appendage (LAA) filling velocity.**

| **Cryptogenic stroke** | **Odds Ratio** | **Std. Err.** | **z** | **P>z** | **[95% Conf.**  **Interval]** |
| --- | --- | --- | --- | --- | --- |
| LAA Filling Velocity | 1.009458 | 0.017832 | 0.53 | 0.594 | 0.975106 – 1.045021 |

LAA: complex left atrial appendage

**Supplemental table 2: Univariate analyses to assess the association between cryptogenic stroke and LAA emptying velocity.**

| **Cryptogenic stroke** | **Odds Ratio** | **Std. Err.** | **z** | **P>z** | **[95% Conf.**  **Interval]** |
| --- | --- | --- | --- | --- | --- |
| LAA Emptying Velocity | 1.028586 | 0.016129 | 1.8 | 0.072 | 0.997455 – 1.060688 |

LAA: complex left atrial appendage

**Supplemental table 3: Multivariate analyses to assess the association between cryptogenic stroke and variables like age, CHA2DS2-VASc score, complex left atrial appendage (LAA) morphology, LAA filling velocity and LAA emptying velocity.**

| **Cryptogenic stroke** | **Odds Ratio** | **Std. Err.** | **z** | **P>z** | **[95% Conf.**  **Interval]** |
| --- | --- | --- | --- | --- | --- |
| Age | 0.851804 | 0.051396 | -2.66 | 0.008 | 0.756798 – 0.958736 |
| CHA2DS2-VASc score | 0.972123 | 0.267423 | -0.1 | 0.918 | 0.566973 – 1.666786 |
| Complex LAA morphology | 3.16193 | 2.647805 | 1.37 | 0.169 | 0.612569–16.32111 |
| LAA Filling Velocity | 1.002691 | 0.030056 | 0.09 | 0.929 | 0.945479 – 1.063365 |
| LAA Emptying Velocity | 1.028391 | 0.023853 | 1.21 | 0.227 | 0.982687 – 1.076221 |

LAA: complex left atrial appendage
