# Supplementary figures and images for: Complex Left Atrial Appendage Morphology Is an Independent Risk Factor for Cryptogenic Ischemic Stroke
Source: Front Cardiovasc Med. 2018 Oct 23;5:131. doi: 10.3389/fcvm.2018.00131 (PMC6232927; doi:10.3389/fcvm.2018.00131)

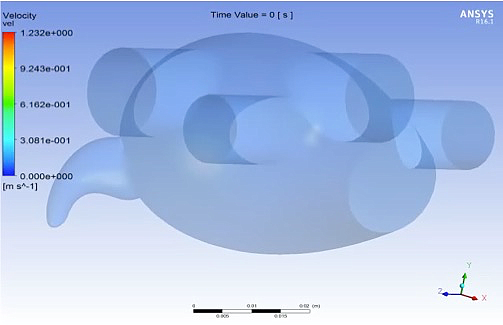

Supplement: Supplemental Image 1 — Flow velocities in simple/chicken wing LAA. [file Image_1.TIFF]

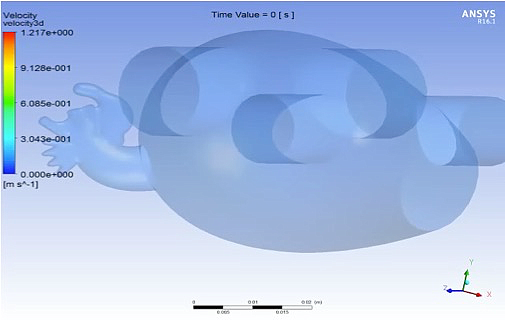

Supplement: Supplemental Image 2 — Flow velocities in complex/non chicken wing LAA. [file Image_2.TIFF]
